# Metabolomics analysis reveals the bitter active ingredients in *Lonicera caerulea* L.

**DOI:** 10.3389/fnut.2025.1650764

**Published:** 2025-09-18

**Authors:** Ling Zhu, Xindi Zhang, Yaxi Han, Kunlun Wang, Xinmiao Yao, Ye Zhou, Bo Li, Nina Ji, Shuwen Lu, Lijun Guan

**Affiliations:** ^1^Institute of Food Processing Research, Heilongjiang Province Academy of Agricultural Sciences, Harbin, China; ^2^Heilongjiang Province Key Laboratory of Food Processing, Harbin, China; ^3^Institute of Soya Research, Heilongjiang Province Academy of Agricultural Sciences, Harbin, China

**Keywords:** blue honeysuckle, sensory evaluation, electronic tongue, bitterness, metabolomics

## Abstract

**Introduction:**

Blue honeysuckle (*Lonicera caerulea* L.) is a nutritionally valuable cold-climate berry characterized by a considerable bitter taste. While bitter compounds in plant foods are often associated with favorable physiological activities, their specific identities in blue honeysuckle remain unclear.

**Methods:**

This study combined sensory evaluation, electronic tongue analysis, and untargeted metabolomics based on ultra-performance liquid chromatography coupled with tandem mass spectrometry (UPLC-MS/MS) to identify and compare bitter compounds in three cultivars of blue honeysuckle.

**Results:**

A total of 73 bitter metabolites were identified in blue honeysuckle, predominantly flavonoids, amino acids and derivatives, phenolic acids, lipids, and tannins. The highest-bitterness variety, Chaoxian (CX) exhibited specific accumulation of l-valine, l-leucine, l-histidine, l-phenylalanine, eriodictyol, trifolin, isoorientin, naringin, and eriocitrin compared to Luohuotan (LHT) and Lanjingling (LJL). The KEGG enrichment analysis implicated the biosynthesis of amino acids (ko01230) and flavonoid biosynthesis (ko00941) as primary contributors to inter-varietal bitterness divergence.

**Discussion:**

These findings provide important information for retaining bioactive bitter metabolites in blue honeysuckle and optimizing its flavor profile to enhance market acceptability.

## 1 Introduction

Blue honeysuckle (*Lonicera caerulea* L.), belonging to Caprifoliaceae family, mainly distributed in cold temperate regions of China, Russia, Japan, and America, is a cold-land small berry with high nutritional and medicinal value (1, 2). The blue honeysuckle fruit is oval-shaped, blue-purple, covered with white frost, and similar in appearance and taste to blueberries (2, 3). As an emerging third-generation fruit, blue honeysuckle is known as “the king of the third generation of small berries” due to its high nutritional and health value (2, 4). Blue honeysuckle has garnered significant attention not only for its unique flavor, but also for its rich content of bioactive compounds, including amino acids, vitamins, phenolic acids, anthocyanins, organic acids, and flavonoids. These bioactive compounds collectively drive its multifaceted health benefits, including antioxidant, anti-inflammatory, hypolipidemic, hepatoprotective, and vasoprotective properties, which underpin its growing scientific and commercial significance (5, 6). As of 2022, the worldwide planting area for this crop was projected to have expanded to around 10,000 hectares (7). As a novel functional small berry, blue honeysuckle has been increasingly introduced into the food market in the form of juices, jams, wines, and cans (6, 8).

The recognition threshold for bitter components is usually low and can even be tasted in micromolar concentrations, making bitter the most easily perceived flavor (9). Despite blue honeysuckle exhibit favorable nutritional qualities, its sensory characteristics, particularly bitterness, pose challenges to consumer acceptance and industrial applications (1). Bitterness, often considered an undesirable flavor, can significantly affect the overall flavor profile of fruit products and influence consumer preference (10). However, bitter compounds often have good physiological activity (11). Previous studies on plant bitterness have mainly focused on compounds such as alkaloids, flavonoids, terpenes, and phenolic acids, which are known contributors to bitter taste in various fruits and vegetables (12–14). Currently, there is a lack of research on specific bitter compounds in blue honeysuckle and their variation between cultivars. Xia et al. (1) identified 7-ketologanin, sweroside, and loganin as key bitter compounds in blue honeysuckle through sensory-guided analysis. The high accumulation of 7-ketologanin was considered the primary factor contributing to the significantly stronger bitterness intensity observed in wild varieties A1 (37.11 ± 0.08 mg/100 g) and A2 (34.70 ± 0.01 mg/100 g), compared to the cultivated varieties ‘Beilei’ (7.04 ± 0.01 mg/100 g), “Vladivostok” (7.05 ± 0.02 mg/100 g), and ‘Lanjingling’ (2.84 ± 0.02 mg/100 g). Zhang et al. (6) found that loganic acid, arbutin, and coumarin were the main components responsible for the differences in bitter flavor among different varieties of blue honeysuckle fruits (Wulan, Berel, and *L. pallasii*). Studies have shown that bitter substances in fruits and vegetables are usually beneficial to human health (15), therefore, identifying bitter components in blue honeysuckle facilitates the enhanced utilization of this berry and supports its industrial-scale applications. Metabolomics has proven to be a powerful tool for elucidating complex biochemical traits in plants, enabling the discovery of key metabolites associated with taste, aroma, and nutritional quality (16, 17).

In the present study, systematic characterization of three different varieties of blue honeysuckle (Chaoxian, Luohuotan, and Lanjingling) was carried out using sensory evaluation, electronic tongue analysis, and untargeted metabolomics. The objectives of this study were to compare the metabolite profiles of three distinct blue honeysuckle varieties (CX, LHT, and LJL), identify potential compounds contributing to bitterness, and provide insights into metabolic pathways associated with the biosynthesis of bitter components in blue honeysuckle. The findings of this study provide valuable information for the future isolation of bitter bioactive compounds in blue honeysuckle and the improvement of the sensory quality of blue honeysuckle-derived food products.

## 2 Materials and methods

### 2.1 Materials and chemicals

**Figure 1:**
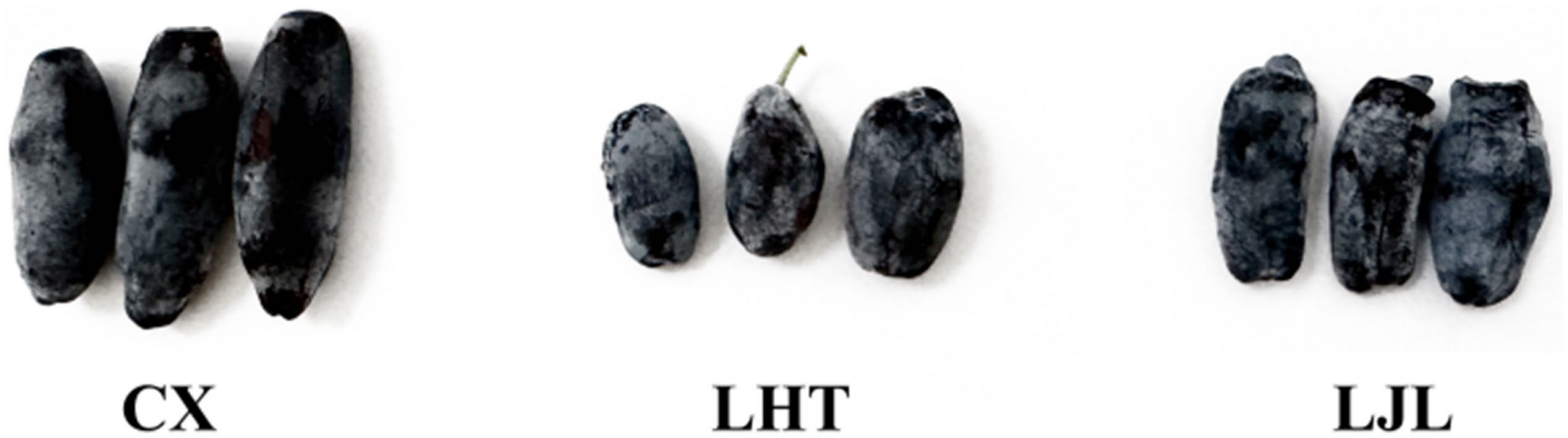
Morphological characterization of three varieties of blue honeysuckle.

Three varieties of blue honeysuckle, namely Chaoxian (CX), Luohuotan (LHT), and Lanjingling (LJL), were selected for the experiment. All blue honeysuckle samples were harvested at full ripeness in mid-June from the same experimental orchard (Harbin, Heilongjiang, China) under consistent cultivation conditions. The three cultivars of blue honeysuckle exhibited distinct morphological differences (Figure 1). “CX” berries exhibitd a slender cylindrical shape, with average fruit length and width of approximately 2.3 cm and 1.3 cm, respectively; ‘LHT’ fruits were shorter, rounder, and oval-shaped, averaging approximately 1.5 cm in length and 0.9 cm in width; “LJL” fruits displayed intermediate length (about 2.1 cm long and 1.0 cm wide) with irregular morphology and slight flattening. Methanol, acetonitrile, and formic acid (chromatographically pure) were purchased from Kemio Chemical Tengda Biotechnology Co. (Xi’an, China). Potassium chloride and tartaric acid (analytical grade) were supplied by Merck KGaA (Darmstadt, Germany). Other reagents used were of analytical grade.

### 2.2 Determination of the chemical constituents of blue honeysuckle samples

#### 2.2.1 Total flavonoid (TF) determination

The content of TF was determined by using the aluminum nitrate colorimetric method (18). Fresh blue honeysuckle fruit samples were homogenized into a uniform pulp. The uniform pulp sample (0.5 g) was extracted twice with 60% ethanol (*v/v*) under reflux (20 mL per extraction). The filtrates were combined, evaporated to dryness, dissolved in 60% ethanol (*v/v*), and diluted to a final volume of 25 mL. The sample solution (1 mL) was mixed with 1 mL of 5% sodium nitrite solution (*m/v*) and allowed to stand for 6 min, then, 1 mL of 10% aluminum nitrate solution (*m/v*) was added and the mixture was left to stand for 6 min. Subsequently, 1 mL of sodium hydroxide solution (4%, *m/v*) and ethanol solution (60%, *v/v*) were added, and the mixture was left to stand for 15 min. An ultraviolet–visible spectrophotometer (T600, Persee General Instrument Co., Ltd., Beijing, China) was employed to measure the absorbance of the mixture at 510 nm. Rutin was selected as the standard in this assay, and the results were expressed as milligrams of rutin equivalents per gram of sample.

#### 2.2.2 Total sugar (TS) determination

The TS content was analyzed using the phenol-sulfuric acid method (19). First, 1.0 g of uniform pulp sample was mixed with 20 mL of distilled water and extracted at 90°C for 3 h. After extraction, the mixture was filtered, and the residue was washed with distilled water. The filtrate and wash solution were combined and diluted with distilled water to a final volume of 250 mL. The diluted test sample (1 mL) was taken, then, 1 mL of 5% phenol (*m/v*) and 5 mL of concentrated sulphuric acid were added. After cooling, the absorbance of the mixed solution was measured at 490 nm. Glucose was served as the standard in this determination, with results reported in milligram glucose equivalents per gram of sample.

#### 2.2.3 Total acid (TA) determination

The TA content was analyzed using the titration assay (20). The water used in this test was carbon dioxide-free or neutral distilled water. The uniform pulp sample (5.0 g) was extracted with 50 mL of distilled water at 80°C for 3 min. The extract was filtered after cooling. The filtrate (20 mL) was titrated with 0.05 mol/L sodium hydroxide as the standard titrant, and phenolphthalein was used as the indicator. The TA was expressed as a percentage of malic acid.

#### 2.2.4 Total polyphenol (TP) determination

The content of TF was determined based on the published Folin–Ciocalteu colorimetric method (21). The uniform pulp sample (1.0 g) was mixed with 20 mL of distilled water and extracted at 100°C for 30 min. The mixture was filtered after cooling, and the filtrate is diluted to 50 mL with distilled water. Then, 1 mL of the test solution was mixed with 1 mL of Folin–Ciocalteu reagent and 3 mL of 7.5% sodium carbonate solution (*m/v*). The mixture was diluted to 10 mL with distilled water, thoroughly mixed, and allowed to develop color at room temperature for 30 min, followed by absorbance measurement at 765 nm. Gallic acid was used for standard calibration, and the results were expressed as milligrams of gallic acid equivalents per gram of sample.

#### 2.2.5 Quantitative analysis of free amino acids

Free amino acid content was measured according to the method described by Yang et al. (22) with slight modifications. Fresh blue honeysuckle samples were frozen at −80°C for 12 h and subsequently freeze-dried in a vacuum freeze dryer (Scientz-100F, Xinzhi Biotechnology Co., Ltd., China) at −50°C under 0.5 MPa for 48 h. Then, the freeze-dried blue honeysuckle samples were ground into powder using a grinder (MM 400, Retsch, Germany) at 30 Hz for 1.5 min and sieved through a 60-mesh screen. The blue honeysuckle powder samples (0.5 g) were mixed with sulfosalicylic acid solution (10 mL, 40 g/L) and extracted for 15 h at 4°C. The extracted suspension was centrifuged at 4025 × g for 10 min in a high-speed centrifuge (3-30 K, SIGMA, Germany), and then the supernatant was evaporated to dryness. After drying, the sample was dissolved in 1 mL of sodium citrate buffer and passed through a 0.22 μm filter membrane (Tianjin Linghang Experimental Equipment Co., Ltd., Tianjin, China). The content of free amino acids was measured by an automatic amino acid analyzer (A300, MembraPure, Germany). The buffer flow rate was 20 mL/h, and the reaction flow rate was 10 mL/h. A sodium-form cationic resin column (200 mm × 4.6 mm) was employed for the test, with a feed volume of 50 μL, and detector wavelengths of 570 nm and 440 nm.

### 2.3 Electronic tongue analysis

Taste attributes of the three different varieties of blue honeysuckle samples were determined using a TS-5000Z electronic tongue system (INSENT Corporation, Japan). Fresh blue honeysuckle fruit samples were squeezed to obtain the juice. For the analysis of sourness, bitterness, astringency, umami, and saltiness, the juice was diluted three times with distilled water. For sweetness analysis, a separate aliquot of the juice was diluted 100 times with distilled water.

### 2.4 Sensory evaluation of bitter taste levels

The sensory panel was composed of 15 members (8 females and 7 males, aged 25–45 years) and underwent specialized bitterness recognition training with quinine solutions (0.01, 0.02, 0.03, and 0.05 g/L). Fresh blue honeysuckle samples from three varieties (CX, LHT, and LJL) were randomly coded and presented to sensory panelists at room temperature (25 ± 1°C). The intensity of bitterness was rated using a structured 9-point scale, where 1 represented “not bitter at all” and 9 indicated “extremely bitter.” Between samples, panelists rinsed their mouths with purified water and waited for 1 min to avoid taste interference.

### 2.5 Metabolomics analysis

#### 2.5.1 Metabolome extraction

Metabolome extraction was performed based on the method described by Liu et al. (14) with slight modifications. The freeze-dried blue honeysuckle powder sample (100 mg) prepared in section 2.2.5 was dissolved in 1.2 mL of 70% (*v/v*) methanol extraction solution. The dissolved samples were left overnight at 4°C, during which time they were vortexed six times to increase the extraction rate. The samples were centrifuged at 16100 × g for 10 min, and the supernatants were filtered through a 0.22 μm microfilter and kept in an injection bottle for Ultra Performance Liquid Chromatography (UPLC)-Tandem mass spectrometry (MS/MS) analysis.

#### 2.5.2 UPLC-MS/MS analysis

The data acquisition instrument system was mainly composed of UPLC (Nexera X2, SHIMADZU, Japan) and MS/MS (4,500 QTRAP, SCIEX, Holland). UPLC conditions: chromatographic column: Agilent SB-C18, 1.8 μm, 2.1 mm × 100 mm; mobile phase A: ultrapure water (containing 0.1% formic acid), mobile phase B: acetonitrile (containing 0.1% formic acid); elution gradient: 0.00 min, 5% B-phase; 0.00–9.00 min, B-phase proportion increased linearly from 5 to 95%; 9.00–10.00 min, 95% B-phase; 10.00–11.10 min, B-phase proportion decreased to 5%; 11.10–14.00 min, 5% B-phase; flow rate: 0.35 mL/min; column temperature: 40°C; injection volume: 4 μL. Mass spectrometry conditions: The electrospray ionization temperature was 550°C, the mass spectrometry voltage was 5,500 V (positive mode)/−4,500 V (negative mode), the curtain gas was 25 psi, and the collision-induced dissociation parameter was set to high. In triple quadrupole mass spectrometers, each ion pair was scanned for detection based on optimized declustering potential and collision energy settings (23).

A self-built metabolite database provided by MetWare Biotechnology Co., Ltd. (Wuhan, China) was used for metabolite identification. The differential analysis of metabolites was performed on the Metware Cloud Platform (MetWare Biotechnology Co., Ltd., Wuhan, China). BitterDB database^1^ was utilized to identify potential bitter metabolites. The identified metabolites were functionally annotated by the Kyoto Encyclopedia of Genes and Genomes (KEGG) Compound database,^2^ followed by topological mapping to KEGG Pathway maps to elucidate their metabolic network relationships.

### 2.6 Data analysis

The results of three parallel tests were averaged and expressed as mean ± standard deviation. Statistical analyses were conducted using IBM SPSS Statistics 22.0 (SPSS Inc., Chicago, IL, United States), with significant differences among groups determined by one-way analysis of variance (ANOVA) followed by Duncan’s multiple range test for *post hoc* pairwise comparisons at *p* < 0.05. Principal component analysis (PCA) and hierarchical cluster analysis cluster were conducted using Origin 2023 (OriginLab Corporation, Northampton, Massachusetts, United States) software, and scientific graphs were generated.

## 3 Results and discussion

### 3.1 Chemical constituents of blue honeysuckle

Flavonoids, sugars, and polyphenols were widely present in blue honeysuckle, as reported in previous studies (2, 8). The contents of TF, TS, TA, and TP of the three varieties of blue honeysuckle were shown in Table 1. Among the three cultivars, CX exhibited the highest contents of TF and TP, followed by LHT with intermediate values, while LJL showed the lowest contents of TF and TP. CX and LHT showed no significant difference in TS content, but both were significantly higher than LJL. In terms of TA content, the level in LHT was significantly higher than those in CX and LJL. These compositional differences are likely to influence the sensory characteristics of the cultivars, particularly the bitterness, as higher TF and TP contents have been associated with increased bitter taste intensity (24).

**Table 1:** The constituents and bitterness intensity of three varieties of blue honeysuckle samples.

Sample	TF (mg/g)	TS (mg/g)	TA (%)	TP (mg/g)
CX	10.5 ± 0.360^a^	51.4 ± 1.37^a^	1.7 ± 0.030^a^	5.1 ± 0.10^a^
LHT	9.70 ± 0.630^b^	48.9 ± 2.27^a^	2.0 ± 0.030^b^	4.6 ± 0.070^b^
LJL	9.04 ± 0.210^c^	41.5 ± 3.38^b^	1.9 ± 0.050^c^	4.3 ± 0.19^c^

The experimental results are expressed as the mean ± standard deviation (*n* = 3). Different letters in the same column indicate a significant difference (*p* < 0.05).

Free amino acids act as key contributors to the sensory characteristics of natural foods, with diverse taste profiles including sourness, sweetness, bitterness, and umami (10). The free amino acid contents of different varieties in blue honeysuckle were shown in Table 2. A complete set of 22 amino acids was measured, consisting of 9 essential amino acids, and 13 non-essential amino acids. Among them, l-glutamine, l-glutamic acid, and l-aspartic acid collectively account for over 80% of the total amino acid content in blue honeysuckle, constituting the primary components of its free amino acid profile. These free amino acids serve as the primary contributors to the umami taste of blue honeysuckle. The contents of l-arginine, l-lysine, l-valine, l-leucine, and l-phenylalanine reached the maximum levels in CX. Based on sensory attributes, the aforementioned amino acids are generally associated with bitterness (22, 25).

**Table 2:** Content of free amino acids in different varieties of blue honeysuckle.

Free amino acid	Amino acid type (essential/non-essential)	Content (μg/g, dry weight)		
		CX	LHT	LJL
l-Histidine	Essential	2.0 ± 0.070^a^	1.2 ± 0.10^b^	0.75 ± 0.11^c^
l-Arginine	Non-essential	46.8 ± 1.56^a^	32.2 ± 2.54^b^	27.5 ± 2.42^c^
l-Asparagine	Non-essential	62.7 ± 4.44^a^	8.72 ± 0.390^b^	12.7 ± 0.29^c^
l-Glutamine	Non-essential	2081 ± 56.59^a^	1761 ± 59.57^c^	1984 ± 19.34^ab^
l-Serine	Non-essential	49.6 ± 0.670^a^	30.8 ± 0.620^b^	32.9 ± 0.250^b^
l-Glycine	Non-essential	6.1 ± 0.29^a^	4.8 ± 1.0^b^	5.2 ± 0.91^b^
l-Aspartic acid	Non-essential	82.5 ± 1.02^a^	82.2 ± 3.05^a^	103 ± 0.570^b^
l-Citrulline	Non-essential	0.6 ± 0.1^a^	0.4 ± 0.07^b^	0.5 ± 0.2^c^
l-Glutamic acid	Non-essential	342 ± 3.39^a^	187 ± 5.72^b^	291 ± 0.930^c^
l-Threonine	Essential	25 ± 0.12^a^	16 ± 0.51^b^	18 ± 0.53^c^
l-Alanine	Non-essential	54 ± 3.2^a^	24 ± 0.80^b^	30 ± 0.23^c^
γ-Aminobutyric acid	Non-essential	67 ± 0.97^a^	41 ± 3.0^b^	42 ± 1.2^b^
l-Proline	Non-essential	66.9 ± 2.47^a^	10.4 ± 1.01^b^	13.0 ± 1.23^c^
l-Ornithine	Non-essential	2 ± 0.04^a^	1 ± 0.03^b^	1 ± 0.03^b^
l-Lysine	Essential	18 ± 0.23^a^	11 ± 0.27^b^	17 ± 0.23^c^
l-Tyrosine	Non-essential	7.9 ± 0.29^a^	7.0 ± 0.25^a^	11 ± 0.88^b^
l-Methionine	Essential	1.1 ± 0.040^a^	1.7 ± 0.090^b^	1.6 ± 0.010^b^
l-Valine	Essential	30 ± 0.42^a^	13 ± 0.25^b^	11 ± 0.45^c^
l-Isoleucine	Essential	4.2 ± 0.010^a^	3.6 ± 0.050^b^	6.7 ± 0.22^c^
l-Leucine	Essential	11 ± 0.11^a^	6.8 ± 0.29^b^	5.9 ± 0.10^c^
l-Phenylalanine	Essential	39 ± 0.59^a^	26 ± 0.80^b^	20 ± 0.96^c^
l-Tryptophan	Essential	9.0 ± 0.48^a^	12 ± 0.47^b^	19 ± 0.10^c^

The experimental results are expressed as the mean ± standard deviation (*n* = 3). Different letters in the same row indicate a significant difference (*p* < 0.05).

### 3.2 Electronic tongue analysis

The taste attributes of three different varieties of blue honeysuckle were measured by the electronic tongue system. Since the reference solution was prepared using 30 mM potassium chloride and 0.3 mM tartaric acid, which contain small amounts of acid and salt, the tasteless points of the sourness and saltiness were −13 and −6, respectively. As shown in Table 3, blue honeysuckle exhibited diverse taste profiles, and all other taste indicators except for richness are effective indicators (with response values higher than the tasteless point). Sourness was the prominent flavour indicator of all three blue honeysuckle fruits, with LHT being the most acidic, which is consistent with the results of the TA measurements. Blue honeysuckle fruits had a certain degree of bitterness and astringency, and there were some differences in bitterness and astringency among the three varieties, with CX having the greatest bitterness, aftertaste-bitterness (B), and aftertaste-astringency (A), LHT being in the middle, and LJL having the least. The bitterness intensity of the three blue honeysuckle varieties (CX, LHT, and LJL) aligns consistently with sensory evaluation test results.

**Table 3:** Determination of electronic tongue taste characteristics of different varieties of blue honeysuckle.

Taste indicator	Tasteless point	CX	LHT	LJL
Sourness	−13	9.4 ± 0.040^a^	10 ± 0.030^b^	10 ± 0.050^b^
Bitterness	0	2.8 ± 0.020^c^	1.3 ± 0.030^b^	1.8 ± 0.020^a^
Astringency	0	1.5 ± 0.020^b^	1.8 ± 0.010^a^	1.3 ± 0.020^c^
Aftertaste-B	0	0.70 ± 0.010^b^	0.39 ± 0.020^a^	0.37 ± 0.020^a^
Aftertaste-A	0	2.1 ± 0.020^c^	1.7 ± 0.020^b^	1.46 ± 0.02^a^
Umami	0	1.8 ± 0.040^b^	1.9 ± 0.040^a^	2.0 ± 0.050^a^
Richness	0	0.08 ± 0.05^c^	−0.4 ± 0.04^a^	−0.2 ± 0.12^b^
Saltiness	−6	1.2 ± 0.020^c^	1.0 ± 0.0040^b^	0.50 ± 0.020^a^
Sweetness	0	4.0 ± 0.040^a^	4.3 ± 0.080^b^	4.4 ± 0.050^b^

Data are expressed as mean ± standard deviation (*n* = 3). Significant differences (*p* < 0.05) are indicated by different superscript letters within a row.

**Figure 2:**
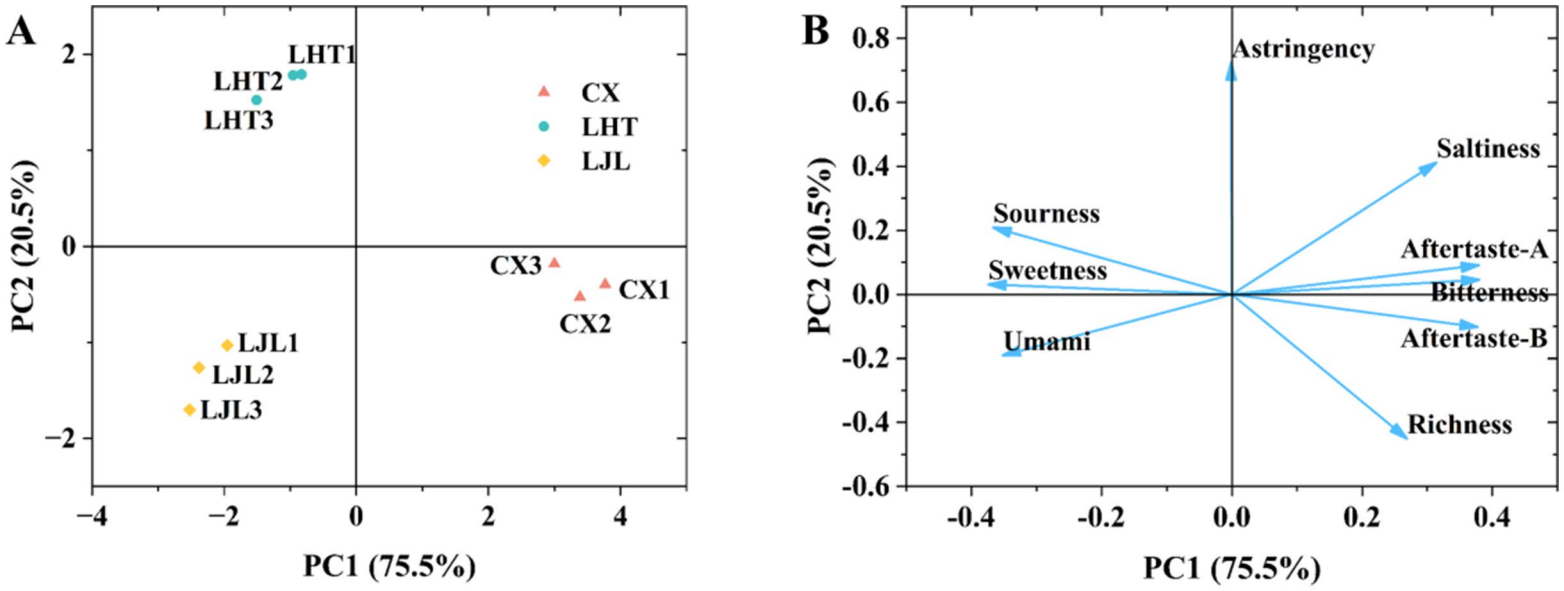
**(A)** Principal component analysis (PCA) of three blue honeysuckle varieties based on electronic tongue data. **(B)** Loadings plot of taste attributes from electronic tongue analysis for blue honeysuckle varieties. The plot illustrates the contribution of each taste attribute to the first two principal components, which together explain 96.0% of the total variance (PC1: 75.5%; PC2: 20.5%). The direction of the vectors indicates the correlation between the original taste attribute and the PCs, while their length represents the magnitude of that contribution.

PCA is a multivariate analytical tool designed to uncover underlying patterns and correlations within complex datasets (26). Based on the PCA results (Figure 2A), the first principal component (PC1) and the second principal component (PC2) explained 75.5 and 20.5% of the variation, respectively. The proximity of the sample points within the group indicated high intra-group reproducibility, while the considerable distance between the sample points of various groups suggested differences in flavor among the three blue honeysuckle groups (27). The taste attribute contribution plot (Figure 2B) demonstrated that bitterness exhibited the highest contribution along the PC1, followed by astringent-A and aftertaste-B. On the PC2, astringency, saltiness, and sourness demonstrated significant contributions. These findings indicated that the flavor differentiation among the three blue honeysuckle varieties was primarily attributed to the aforementioned taste attributes.

### 3.3 Bitter taste levels evaluation of three varieties of blue honeysuckle

**Figure 3:**
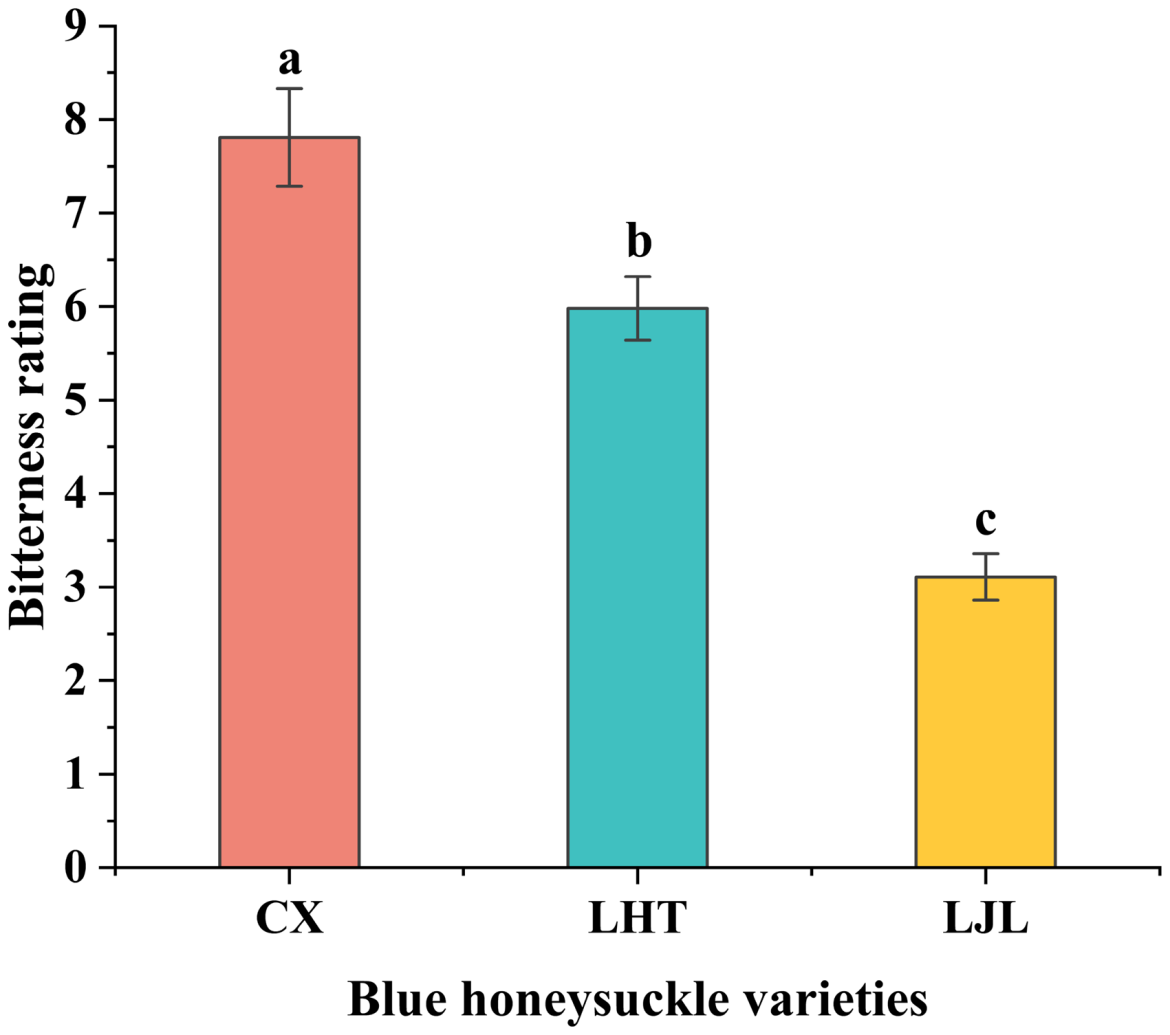
Bitter taste levels of three blue honeysuckle cultivars. Data represent mean ± standard deviation of three repeats. Different letters above the bars indicate significant differences.

Results from sensory evaluation confirmed significant differences in bitter taste intensity among the three varieties (Figure 3), with CX showing the highest average bitterness score of 7.8 ± 0.52, followed by LHT with a moderate bitterness score of 6.0 ± 0.34, and LJL recording the lowest bitterness score of 3.1 ± 0.25.

### 3.4 Metabolic profiles and differential metabolites in three varieties of blue honeysuckle

A total of 692 metabolites were identified, including 71 amino acids and their derivatives, 105 phenolic acids, 36 nucleotides and their derivatives, 176 flavonoids, 2 quinones, 26 lignans and coumarins, 11 tannins, 9 alkaloids, 23 terpenes, 65 organic acids, 104 lipids, and 64 other metabolites. Among them, flavonoids, phenolic acids, and lipids were established as the principal constituents of CX, LHT, and LJL. These functional compounds demonstrated antioxidative properties by mitigating oxidative stress and suppressing *β*-amyloid production, thereby indirectly attenuating Alzheimer’s disease (14).

**Figure 4:**
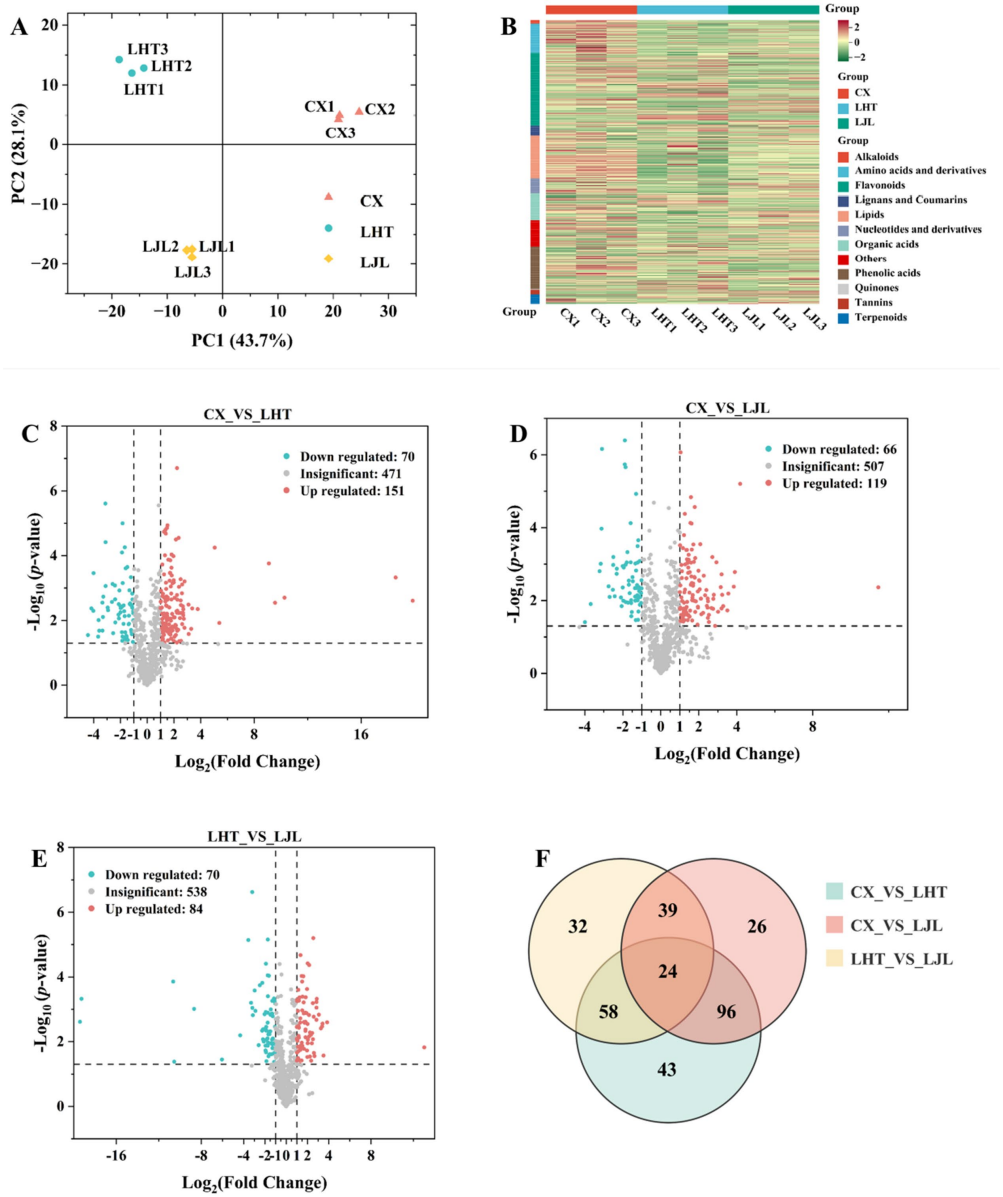
**(A)** PCA analysis of all metabolite profiles across different blue honeysuckle groups. **(B)** Hierarchical clustering heatmap of all metabolites in blue honeysuckle of three varieties. Red indicates a high relative content and green indicates a low relative content. **(C–E)** Volcano plot of differentially accumulated metabolites in blue honeysuckle with *p* ≤ 0.05 and |log_2_FC| ≥ 1 between **(C)** CX vs. LHT, **(D)** CX vs. LJL, **(E)** LHT vs. LJL. The red, green, and gray dots represent the up-regulated, down-regulated, and non-significantly differentially accumulated metabolites between the comparison groups, respectively. **(F)** Venn diagram of differentially accumulated metabolites among CX, LHT, and LJL.

PCA of metabolite data was carried out for three varieties of blue honeysuckle samples. As shown in Figure 4A, the contribution rates of PC1 and PC2 were 43.7 and 28.1%, respectively, with a combined total of 71.8%, suggesting that PC1 and PC2 contained most of the metabolite information of the blue honeysuckle samples (26). The three blue honeysuckle varieties (CX, LHT, and LJL) were clearly separated and the three biological replicate samples of each variety were clustered together, demonstrating the reliability and good repeatability of the experiment, as well as significant differences among the three varieties (28). Moreover, distinct clustering into three groups was evident on the hierarchical clustering heatmap (Figure 4B), indicating significant differences in metabolites among the three varieties.

To identify metabolites with significant differences in accumulation between blue honeysuckle varieties, a screening threshold of |log_2_Fold change (FC)| ≥ 1 and *p* ≤ 0.05 was applied. As shown in Figure 4C, a total of 221 differential metabolites were detected between CX and LHT, with 151 up-regulated and 70 down-regulated metabolites in CX. Among the 185 differential metabolites identified between CX and LJL, 119 and 66 metabolites were up-regulated and down-regulated in CX, respectively (Figure 4D). In addition, 154 differential metabolites were present between LHT and LJL, with 84 and 70 metabolites up- and down-regulated in LHT, respectively (Figure 4E). The differential metabolites in various blue honeysuckle samples could be categorized into more than 11 classes (Table 4), and the majority of differential metabolites were categorized into four classes, including flavonoids, amino acids and derivatives, lipids, and phenolic acids. Meanwhile, multiple comparative analysis (Figure 4F) revealed that three different varieties of blue honeysuckle had 24 shared differential metabolites. These metabolites covered multiple biochemical classes, mainly flavonoids (e.g., isohyperoside, pinocembrin-7-O-neohesperidoside, and kaempferol-3,7-di-O-glucoside), amino acids and derivatives (e.g., 5-hydroxy-l-tryptophan, l-phenylalanine, and l-isoleucine), phenolic acids (e.g., brevifolin carboxylic acid and 3,4,5-tricaffeoylquinic acid), and organic acids (e.g., fumaric acid and succinic anhydride).

**Table 4:** Statistical analysis of differential cumulative metabolite counts for three different varieties of blue honeysuckle.

Metabolite class	CX_vs_LHT		CX_vs_LJL		LHT_vs_LJL	
	Up	Down	Up	Down	Up	Down
Flavonoids	37	31	43	34	37	21
Amino acids and derivatives	24	3	22	4	4	9
Lipids	34	1	15	0	4	12
Phenolic acids	28	8	14	6	14	9
Organic acids	9	10	7	4	11	1
Terpenoids	6	5	7	2	3	3
Alkaloids	2	1	0	1	0	0
Lignans and coumarins	3	4	3	2	4	3
Nucleotides and derivatives	6	2	3	6	0	4
Tannins	0	0	0	5	3	3
Others	12	2	5	2	3	4
Total	151	70	119	66	84	70

Overall, the differentially accumulated metabolites could be broadly divided into two groups (Table 4). The first group of differential metabolites constituted antioxidant components such as flavonoids, alkaloids, phenolic acids, and terpenoids, while the second group included taste-related components such as organic acids, and amino acids and derivatives (29). Most differentially accumulated metabolites of the antioxidant components were found at higher concentrations in CX sample. CX, LHT, and LJL displayed similar metabolite accumulation patterns. There were significant differences in about 20–30% of the metabolites between the various varieties of blue honeysuckle samples, with flavonoids being the major differential metabolites.

### 3.5 Screening and differential analysis of potential bitter metabolites among different varieties of blue honeysuckle

The metabolites identified in the blue honeysuckle samples were further analyzed in comparison with the BitterDB database (30) and relevant literature for further screening of potential bitter metabolites. As shown in Table 5, a total of 73 were screened as potentially associated with the bitterness of blue honeysuckle, including 11 amino acids and derivatives, 11 phenolic acids, 1 nucleotide and derivative, 30 flavonoids, 6 tannins, 2 terpenoids, 1 organic acid, 6 lipids, and 5 other categories. Flavonoids, amino acids and derivatives, phenolic acids, and lipids constituted the most abundant classes of potential bitterness-associated metabolites identified in blue honeysuckle.

**Table 5:** Potential metabolites associated with bitterness detected by UPLC–MS/MS.

Metabolites classification	Metabolites	Formula	Peak area (× 10^4^)			Bitter recognition threshold (mmol/L)	Bitter receptors targets	References
			CX	LHT	LJL			
Flavonoids	Chrysin	C_15_H_10_O_4_	1.7 ± 0.41	1.1 ± 0.089	0.96 ± 0.016	—	hTas2r14, hTas2r39	(32)
	Pinocembrin	C_15_H_12_O_4_	1.53 ± 0.175	1.46 ± 0.190	1.85 ± 0.813	—	hTas2r14, hTas2r39	(32)
	Naringenin	C_15_H_12_O_5_	41.3 ± 3.69	54.8 ± 12.9	96.1 ± 5.52	—	hTas2r14, hTas2r39	(32)
	Phloretin	C_15_H_14_O_5_	23.1 ± 5.62	20.1 ± 2.55	21.4 ± 2.40	—	hTas2r14, hTas2r39	(32)
	Luteolin	C_15_H_10_O_6_	6.35 ± 0.592	4.97 ± 1.04	5.59 ± 0.620	—	hTas2r14, hTas2r39	(32)
	Eriodictyol	C_15_H_12_O_6_	66.7 ± 2.24	18.9 ± 3.36	47.0 ± 4.49	—	hTas2r14, hTas2r39	(32)
	Catechin	C_15_H_14_O_6_	447 ± 17.1	198 ± 22.2	417 ± 44.3	0.41	hTas2r14, hTas2r39	(31, 32)
	Epicatechin	C_15_H_14_O_6_	513.8 ± 10.54	500.6 ± 62.29	1,649 ± 108.9	0.54	hTas2r4, hTas2r14, hTas2r39, mTas2r105	(31, 32, 41)
	Epigallocatechin	C_15_H_14_O_7_	6.38 ± 0.911	8.72 ± 0.206	7.27 ± 1.34	0.52	hTas2r14, hTas2r39	(31, 32)
	Isorhamnetin	C_16_H_12_O_7_	0.92 ± 0.18	0.41 ± 0.16	0.43 ± 0.066	—	hTas2r14, hTas2r39	(32)
	Apigenin-8-C-Glucoside (Vitexin)	C_21_H_20_O_10_	10 ± 0.74	7.4 ± 1.5	3.2 ± 0.13	0.001		(42)
	Apigenin-6-C-glucoside (Isovitexin)	C_21_H_20_O_10_	19.3 ± 1.92	15.3 ± 2.40	7.11 ± 1.16	—		(43)
	Naringenin-7-O-glucoside (Prunin)	C_21_H_22_O_10_	910 ± 85.0	451 ± 54.8	384 ± 21.8	—		(43)
	Kaempferol-3-O-glucoside (Astragalin)	C_21_H_20_O_11_	47.5 ± 8.62	21.5 ± 2.56	31.7 ± 10.5	0.029		(44)
	Quercetin-3-O-rhamnoside (Quercitrin)	C_21_H_20_O_11_	17.7 ± 1.74	9.78 ± 2.30	10.6 ± 0.370	—		(43)
	Kaempferol-3-O-galactoside (Trifolin)	C_21_H_20_O_11_	1946 ± 131.9	519.7 ± 58.30	492.3 ± 36.06	0.00067		(31)
	Luteolin-6-C-glucoside (Isoorientin)	C_21_H_20_O_11_	23 ± 1.8	4.8 ± 0.42	1.5 ± 0.40	0.016		(42)
	Cyanidin-3-O-glucoside (Kuromanin)	C_21_H_21_O_11_	474 ± 9.88	393 ± 7.22	388.1 ± 24.4	—		(45)
	Quercetin-3-O-galactoside (Hyperin)	C_21_H_20_O1_2_	435 ± 36.0	791 ± 94.1	352 ± 42.7	0.004		(42)
	Quercetin-3-O-glucoside (Isoquercitrin)	C_21_H_20_O_12_	439 ± 22.2	858 ± 36.7	389 ± 20.0	0.028		(25)
	Kaempferol-3-O-(6″-malonyl) glucosie	C_24_H_22_O_14_	3.7 ± 0.37	36.4 ± 5.13	12.8 ± 0.881	0.035		(44)
	Apigenin-7-O-neohesperidoside (Rhoifolin)	C_27_H_30_O_14_	234 ± 42.2	135 ± 21.2	42.4 ± 1.88	0.029		(42)
	Naringenin-7-O-Rutinoside (Narirutin)	C_27_H_32_O_14_	390 ± 13.6	76.6 ± 8.10	207 ± 28.7	0.042		(42)
	Naringenin-7-O-Neohesperidoside (Naringin)	C_27_H_32_O_14_	446 ± 43.5	69.9 ± 7.11	185 ± 14.6	0.01		(44)
	Kaempferol-3-O-rutinoside (Nicotiflorin)	C_27_H_30_O_15_	493.1 ± 24.05	303.5 ± 10.01	1,132 ± 213.4	0.00043		(31)
	Eriodictyol-7-O-Rutinoside (Eriocitrin)	C_27_H_32_O_15_	75.8 ± 14.4	17.2 ± 4.08	41.4 ± 3.66	0.012		(42)
	Diosmetin-7-O-rutinoside (Diosmin)	C_28_H_32_O_15_	343 ± 207	587 ± 26.0	322 ± 34.9	0.023		(42)
	Diosmetin-7-O-Neohesperidoside (Neodiosmin)	C_28_H_32_O_15_	157 ± 105	80.7 ± 9.02	134 ± 12.2	0.022		(42)
	Quercetin-3-O-rutinoside (Rutin)	C_27_H_30_O_16_	150 ± 3.06	145 ± 25.9	182.0 ± 7.5	0.117		(42)
	Isorhamnetin-3-O-rutinoside (Narcissin)	C_28_H_32_O_16_	1,105 ± 51.03	690.3 ± 52.88	1,288 ± 263.4	0.033		(42)
Amino acids and derivatives	l-Valine	C_5_H_11_NO_2_	2,684 ± 274.8	737.5 ± 23.37	649.5 ± 49.20	30		(36)
	l-Leucine	C_6_H_13_NO_2_	1,652 ± 215.7	398.2 ± 39.85	381.8 ± 24.30	12		(36)
	l-Isoleucine	C_6_H_13_NO_2_	3,807 ± 641.0	1,020 ± 96.67	980.5 ± 78.01	10		(37)
	l-Lysine	C_6_H_14_N_2_O_2_	261 ± 39.4	40.2 ± 11.2	46.7 ± 4.10	80		(37)
	l-Histidine	C_6_H_9_N_3_O_2_	31 ± 6.4	4.5 ± 1.0	3.4 ± 0.70	48	hTas2r1	(36)
	l-Phenylalanine	C_9_H_11_NO_2_	564 ± 153	237 ± 66.7	250 ± 31.8	45	hTas2r1	(37)
	l-Arginine	C_6_H_14_N_4_O_2_	149 ± 63.7	56.5 ± 46.8	46.1 ± 15.5	75		(37)
	l-Tyrosine	C_9_H_11_NO_3_	396 ± 97.4	259 ± 110	379 ± 64.7	4		(37)
	Glycyl-l-leucine	C_8_H_16_N_2_O_3_	4.33 ± 2.01	1.76 ± 0.135	2.21 ± 0.173	22.909	hTas2r1	(46)
	l-Tryptophan	C_11_H_12_N_2_O_2_	24.4 ± 7.24	22.8 ± 11.0	17.9 ± 8.34	5	hTas2r4, hTas2r39	(37)
	l-Glycyl-l-phenylalanine	C_11_H_14_N_2_O_3_	2.5 ± 1.3	1.1 ± 0.34	1.3 ± 0.44	4.365	hTas2r1	(46)
Lipids	Palmitic acid	C_16_H_32_O_2_	3.2 ± 0.46	2.7 ± 0.15	2.8 ± 0.060	0.807		(47)
	α-Linolenic acid	C_18_H_30_O_2_	2,954 ± 196.0	1,151 ± 57.04	2,457 ± 868.3	0.28		(44)
	Linoleic acid	C_18_H_32_O_2_	2,205 ± 283.8	1,615 ± 145.0	1714 ± 572.8	0.93		(40)
	Stearic acid	C_18_H_36_O_2_	3,065 ± 193.2	3,302 ± 376.0	3,059 ± 223.5	0.726		(37)
	(9Z,11E)-13-hydroxyoctadeca-9,11-dienoic acid	C_18_H_32_O_3_	8.4 ± 1.2	6.6 ± 2.2	6.5 ± 0.72	0.79		(40)
	12,13-dihydroxy-9Z-octadecenoic acid	C_18_H_34_O_4_	1.2 ± 0.39	1.0 ± 0.017	1.0 ± 0.043	—		(48)
Nucleotides and derivatives	Adenosine	C_10_H_13_N_5_O_4_	264 ± 121	789 ± 61.6	741 ± 4.29	77		(25)
Tannins	Procyanidin B1	C_30_H_26_O_12_	858 ± 134	159 ± 114	891 ± 80.3	0.4	hTas2r5, hTas2r7	(38, 44)
	Procyanidin B2	C_30_H_26_O_12_	324.3 ± 8.284	331.5 ± 46.72	3,022 ± 186.2	0.485		(49)
	Procyanidin B3	C_30_H_26_O_12_	91.9 ± 5.40	390 ± 22.7	175 ± 40.1	0.5		(44)
	Procyanidin B4	C_30_H_26_O_12_	22.9 ± 2.31	20.7 ± 3.43	20.5 ± 11.8	—	hTas2r5	(38)
	Procyanidin C1	C_45_H_38_O_18_	38.3 ± 2.59	92.2 ± 26.6	25.8 ± 25.2	0.4	hTas2r5	(31, 44)
	Procyanidin C2	C_45_H_38_O_18_	37.4 ± 6.12	118 ± 29.7	215 ± 26.0	0.00104	hTas2r5	(38)
Terpenoids	Sweroside	C_16_H_22_O_9_	116 ± 5.48	510 ± 41.0	658 ± 28.6	—		(50)
	Swertiamarin	C_16_H_22_O_10_	9.49 ± 2.08	12.5 ± 2.69	13.8 ± 4.40	—		(51)
Phenolic acids	Benzamide	C_7_H_7_NO	6.1 ± 0.65	15 ± 5.4	11 ± 1.3	—	hTas2r14	(52)
	5-Hydroxymethylfurfural	C_6_H_6_O_3_	40.7 ± 1.66	40.6 ± 3.84	45.0 ± 3.69	—		(53)
	Salicylic acid	C_7_H_6_O_3_	5.1 ± 0.85	9.8 ± 2.5	8.2 ± 1.6	—	hTas2r14, mTas2r135	(41)
	4-Hydroxybenzoic acid	C_7_H_6_O_3_	11 ± 3.7	11 ± 1.5	5.9 ± 0.43	0.0145		(38)
	Vanillin	C_8_H_8_O_3_	6.24 ± 1.17	7.81 ± 1.55	6.61 ± 2.03	—	hTas2r14, hTas2r39, hTas2r49	(54)
	Gentisic acid	C_7_H_6_O_4_	46.7 ± 16.7	18.2 ± 2.48	24.1 ± 4.33	0.0129		(44)
	Vanillic acid	C_8_H_8_O_4_	4.0 ± 1.9	1.4 ± 0.72	1.6 ± 0.33	0.05947	hTas2r14	(44)
	Caffeic acid	C_9_H_8_O_4_	193 ± 157	149 ± 67.2	38.9 ± 14.4	0.0111	hTas2r1, hTas2r14	(44)
	Arbutin	C_12_H_16_O_7_	78.2 ± 7.24	17.6 ± 14.4	43.3 ± 2.76	0.9	hTas2r16, mTas2r126, oaTas2r811, btTas2r16, laTas2r16c	(25, 52, 55)
	Neochlorogenic acid	C_16_H_18_O_9_	120 ± 2.96	134 ± 32.4	87.3 ± 8.22	—		(56)
	Protocatechuic acid	C_7_H_6_O_4_	103 ± 37.5	44.0 ± 4.88	60.8 ± 2.49	0.0324	hTas2r14, hTas2r47	(44, 57)
Organic acids	Quinic acid	C_7_H_12_O_6_	604 ± 21.8	536 ± 56.3	579 ± 14.0	0.052		(44)
Others	Vitamin B3	C_6_H_5_NO_2_	11.8 ± 7.66	6.21 ± 5.42	9.02 ± 4.34	5.5		(38)
	D-Pantothenic acid	C_9_H_17_NO_5_	121.6 ± 10.94	199.4 ± 16.97	197.7 ± 13.63	—	hTas2r14, hTas2r40, hTas2r43, hTas2r44	(41)
	Resveratrol	C_14_H_12_O_3_	5.82 ± 2.11	5.72 ± 1.28	7.05 ± 1.35	0.206	hTas2r1, hTas2r14, hTas2r39	(32, 38)
	Vitamin B1	C_12_H_17_ClN_4_OS	13 ± 0.39	24 ± 1.2	14 ± 1.3	0.1	hTas2r1, hTas2r7, hTas2r39	(58, 59)
	Vitamin B2	C_17_H_20_N_4_O_6_	13 ± 2.0	4.8 ± 1.2	4.5 ± 0.85	0.65		(38)

Peak area results are expressed as the mean ± standard deviation of three repeats. “—” indicates that no results were found.

Flavonoids have been systematically characterized as the primary bitter principles in fruits and vegetables (13). Catechin and gallic acid are the main bitter substances in green tea, and catechin, epicatechin, gallic acid, and quercetin serve as key contributors to the increased bitterness in wine (10). According to Table 5, the most diverse types of bitter substances screened from blue honeysuckle were flavonoids (30 species), primarily including chrysin, pinocembrin, naringenin, phloretin, luteolin, eriodictyol, catechin, epicatechin, epigallocatechin, isorhamnetin, apigenin-O-glycosides, naringenin-O-glycosides, kaempferol-O-glycosides, quercetin-O-glycosides, luteolin-O-glycosides, cyanidin-O-glycosides, eriodictyol-O-glycosides, diosmetin-O-glycosides, and isorhamnetin-O-glycosides. Xia et al. (1) similarly identified various flavonoid bitter substances such as anthocyanins, flavan-3-ols, flavanonols, flavones, and flavonols in blue honeysuckle. Epigallocatechin is a common catechin compound found in plant-based foods such as tea, apples, and grapes (31). Catechin is the main component of tea polyphenols, accounting for approximately 75 to 80% of the total polyphenol content in tea (32). The above two substances are one of the primary sources of the bitter and astringent taste of tea. Compared with LJL, epicatechin was significantly down-regulated in CX and LHT (Supplementary Table S1). It has been reported that phenolic acids exhibit a sour and astringent taste, whereas quercetin-3-O-rutinoside has a mild astringent taste. Phenolic acids and quercetin-3-O-rutinoside could enhance the bitterness of catechins (33). Most of the flavonoid bitter metabolites exhibited high bitter intensity. For instance, kaempferol-3-O-galactoside and kaempferol-3-O-rutinoside demonstrated remarkably low bitterness thresholds of 0.00067 mmol/L and 0.00043 mmol/L, respectively, both significantly lower than the widely used bitter reference compound quinine hydrochloride (0.3 mmol/L) (22). The content of eriodictyol, isorhamnetin, kaempferol-3-O-galactoside, luteolin-6-C-glucoside, naringenin-7-O-neohesperidoside, and eriodictyol-7-O-rutinoside showed up-regulated expression in CX (Supplementary Table S1). Thus, these flavonoids may be one of the contributing factors to the intensified bitter taste in the varieties of blue honeysuckle.

Amino acids, particularly essential amino acids, frequently exhibit bitter taste profiles and serve as biosynthetic precursors for bitter constituents in food systems (34). Studies have revealed that l-phenylalanine is a key contributor to the bitter taste in bamboo shoots, and l-valine, l-isoleucine, and l-phenylalanine are identified as primary bitter components in *Zanthoxylum Bungeanum* Maxim (22, 35). In blue honeysuckle, several bitter amino acids have been identified, including l-valine, l-leucine, l-isoleucine, l-tyrosine, and l-tryptophan. These amino acids possess relatively high bitterness intensities, with reported taste thresholds of 30, 12, 10, 4, and 5 mmol/L, respectively (36, 37). Compared to the LJL group, l-histidine, l-leucine, l-valine, and l-phenylalanine exhibited significant up-regulation in both CX and LHT samples (FC ≥ 2). The shared differential metabolites between CX, LHT, and LJL included two bitter compounds, l-phenylalanine and l-isoleucine (Figure 4F), which might be the primary contributors to the bitterness of the blue honeysuckle. The potential role of amino acid bitter metabolites in blue honeysuckle bitterness needs to be further explored.

Phenolic acids contribute to bitterness and astringency in wine, peas, and corn germ protein flour (38). In the present study, phenolic acids like benzamide, salicylic acid, gentisic acid, caffeic acid, and arbutin contributed to the bitter flavor of blue honeysuckle. Salicylic acid frequently accumulates in bitter vegetables and fruits such as bitter orange and bitter gourd, but it typically functions as a precursor metabolite or byproduct of the primary bitter components, rather than serving as a direct contributor to bitterness (39). Coffee acid contributes to both the bitterness and astringency of peas (38). Arbutin is a phenolic glycoside compound with a reported bitter taste threshold of only 0.9 mmol/L, which produces a bitter taste by stimulating the hTAS2R16 receptor (25). Fatty acids have recently been identified as the primary bitter compounds in oat and pea protein isolates (37, 40). Blue honeysuckle contained free fatty acids with strong bitterness (bitter recognition threshold < 1 mmol/L), such as palmitic acid, *α*-linolenic acid, linoleic acid, stearic acid, and (9Z,11E) − 13-hydroxyoctadeca-9,11-dienoic acid. Among them, α-linolenic acid, linoleic acid, and stearic acid were more abundant in blue honeysuckle (peak area > 1 × 10^7^). The phenolic acid and fatty acid metabolites showed no significant differences among CX, LHT, and LJL (Supplementary Table S1).

Tannins are oligomers or polymers composed of derivates from (+)-catechin and its isomers, which may be responsible for bitter and astringent flavors in grapes and wine (38). Six bitter tannins, namely procyanidin B1, B2, B3, B4, C1, and C2, were identified in blue honeysuckle. Procyanidin B4 and C2 are characterized with astringent and bitter taste in an aqueous ethanol solution at a concentration of 0.9 g/L. Tannin-derived bitter metabolites identified in blue honeysuckle possess exceedingly low bitterness thresholds (< 0.5 mmol/L). Procyanidin B2, B4, and C1 showed down-regulation in CX and LHT compared to the low-bitter LJL (Supplementary Table S1). In addition to the above substances, a number of terpenoids (sweroside, swertiamarin), vitamins (D-pantothenic acid, vitamin B1, B2, B3), resveratrol, adenosine, and quinic acid with a bitter flavor have been identified in blue honeysuckle. Compared with LHT, vitamin B1 was significantly up-regulated in CX, while sweroside was significantly down-regulated in CX.

### 3.6 Analysis of the biosynthetic pathways of bitter compounds in blue honeysuckle

**Figure 5:**
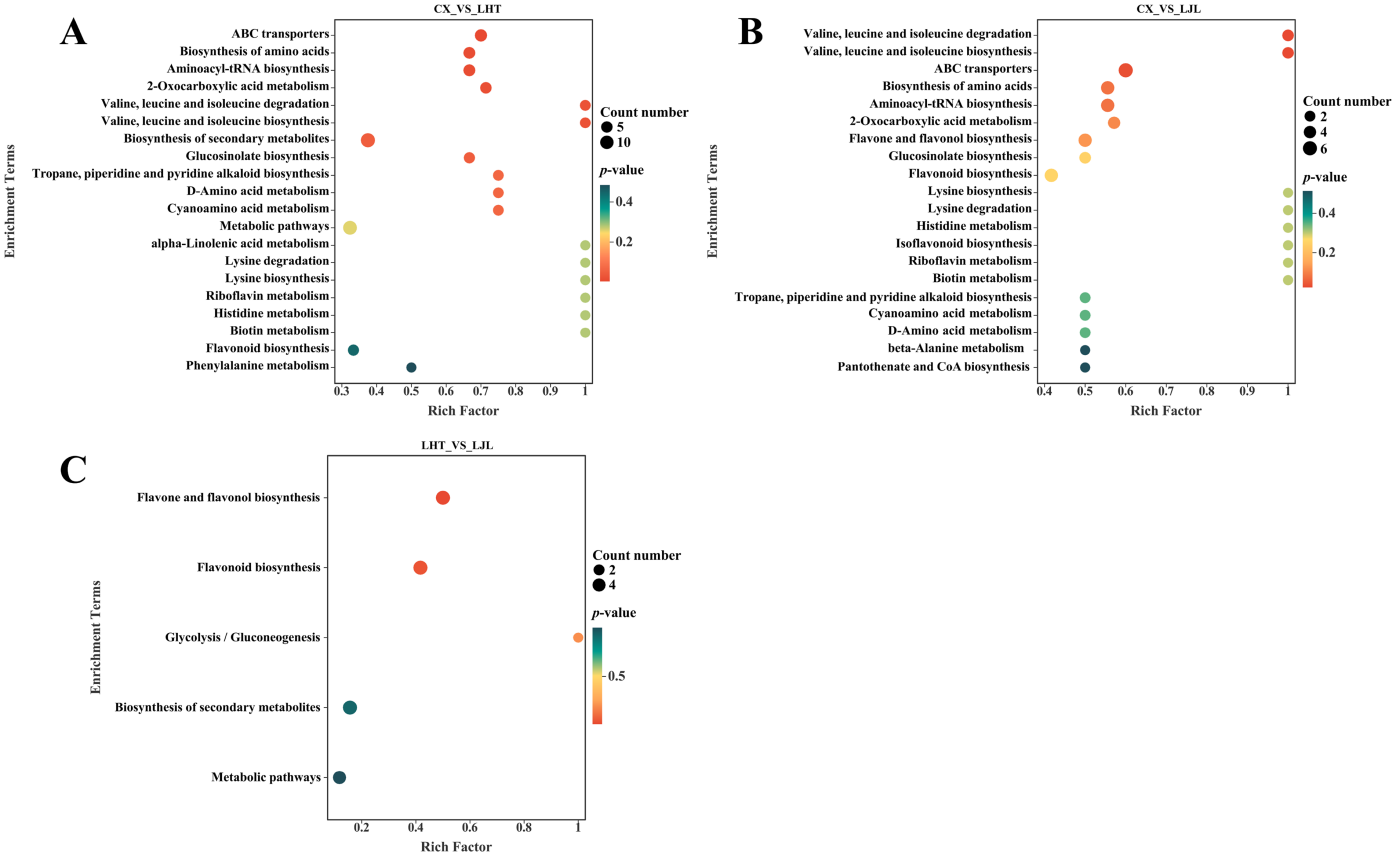
KEGG pathway analysis of differentially bitter metabolites in blue honeysuckle between **(A)** CX vs. LHT, **(B)** CX vs. LJL, **(C)** LHT vs. LJL.

**Figure 6:**
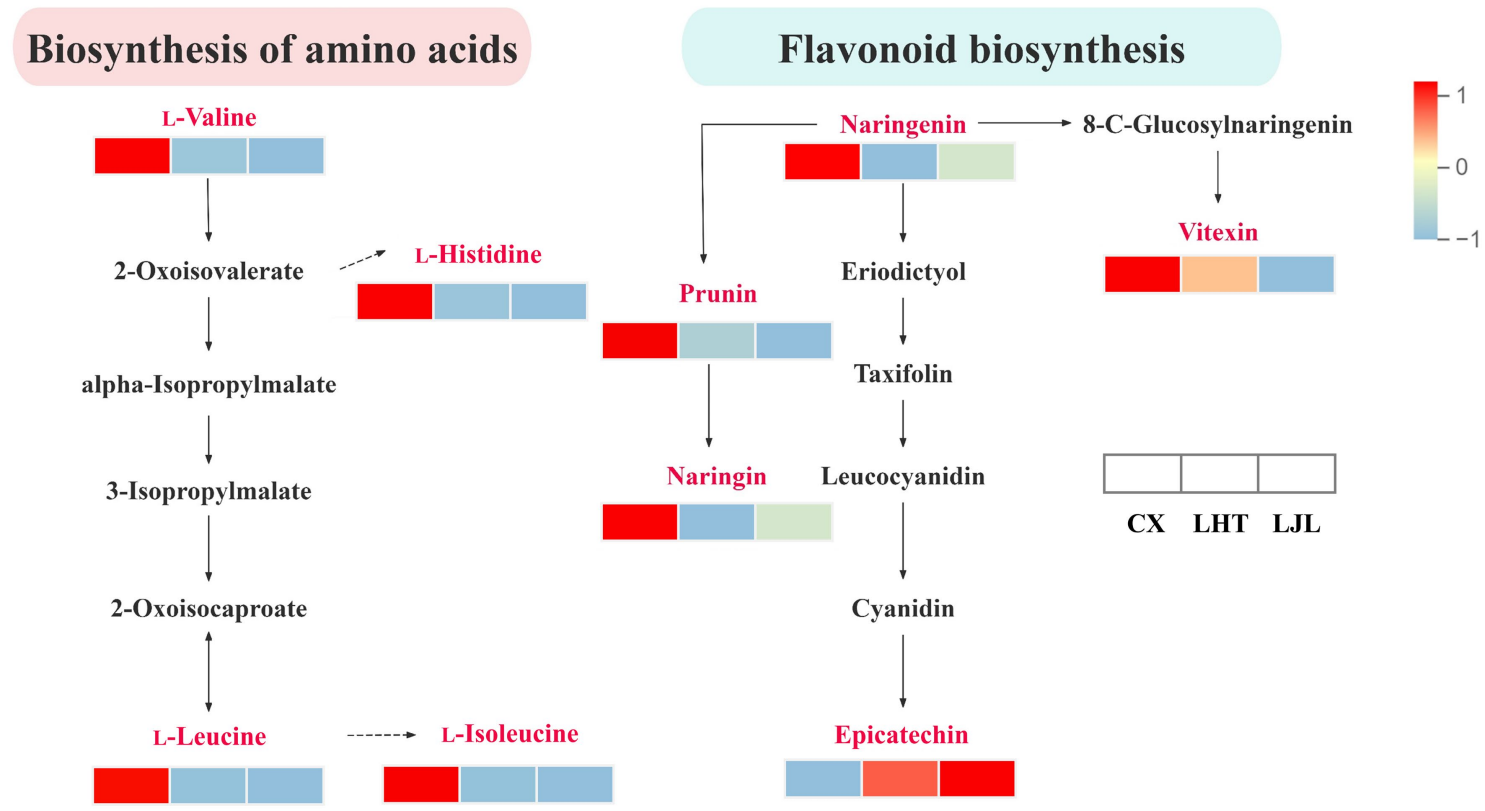
Differences in the biosynthesis of amino acids and flavonoid biosynthesis pathway bitter metabolites among CX, LHT, and LJL. The rectangles represent different varieties of blue honeysuckle. Colors are based on accumulation levels of metabolites, where red represents high accumulation and blue represents low accumulation.

The KEGG database is widely utilized for investigating signal transduction pathways and metabolite accumulation, serving as a principal public repository for pathway analysis (60). As illustrated in Figure 5, the differential bitter metabolites among various blue honeysuckle samples are predominantly accumulated in biosynthesis of secondary metabolites (ko01110), metabolic pathways (ko01100), biosynthesis of amino acids (ko01230), flavonoid biosynthesis (ko00941), aminoacyl-tRNA biosynthesis (ko00970), flavone and flavonol biosynthesis (ko00944), and ABC transporters (ko02010). These enriched KEGG pathways were mostly closely related to fruit growth, development, and flavor formation of blue honeysuckle (13). The biosynthesis of amino acids and flavonoids biosynthesis were identified as key pathways associated with bitter compound accumulation in blue honeysuckle (Figure 6). Within the biosynthesis of amino acids pathway, significant accumulation of branched-chain amino acids (BCAAs), such as l-valine, l-leucine, and l-isoleucine, was observed in the high-bitter cultivar CX compared to LHT and LJL. These amino acids and their metabolic intermediates (e.g., 2-oxoisovalerate, 3-isopropylmalate) are known precursors for various secondary metabolites, including bitter-tasting compounds. The elevated levels of l-histidine in CX also suggest its potential role in bitterness perception. More importantly, the flavonoid biosynthesis pathway showed a strong association with bitterness differentiation. In particular, naringenin, prunin, naringin, and vitexin were significantly up-regulated in CX and LHT, with much lower levels detected in LJL. Epicatechin was found enriched in all three cultivars (peak area > 1 × 10^6^, Table 5) but showed the highest accumulation in LJL, implying that it may contribute to overall taste complexity but is not the primary driver of bitterness. Collectively, the accumulation patterns of l-valine, l-leucine, l-isoleucine, l-histidine, naringin, and vitexin were consistent with the bitterness gradient (CX > LHT > LJL), indicating that they are potential key factors contributing to the bitterness of blue honeysuckle. These compounds might synergistically cause bitter taste variations among blue honeysuckle varieties and warrant further validation as biomarkers of bitterness in future breeding and quality control strategies.

## 4 Conclusion

In summary, this study combined sensory evaluation and non-targeted metabolomics approachs to reveal key bitter compounds and pathways responsible for the bitterness differences among three blue honeysuckle cultivars. Flavonoids and amino acids and derivatives were identified as the major contributors to the bitterness of blue honeysuckle, especially l-valine, l-leucine, l-isoleucine, l-histidine, and vitexin. Enrichment of these compounds in amino acid and flavonoid biosynthesis pathways critically defines CX’s intense bitterness profile. These findings provide valuable insights for improving flavor quality while preserving bioactive components of blue honeysuckle, and have potential applications in functional food development and breeding strategies.

## Data Availability

The original contributions presented in the study are included in the article/Supplementary material, further inquiries can be directed to the corresponding author.

## Footnotes

^1^ https://bitterdb.agri.huji.ac.il/dbbitter.php

^2^ https://www.kegg.jp/kegg/compound/
